# New York Odontological Society

**Published:** 1893-01

**Authors:** 

**Affiliations:** New York Odontological Society


### Reports of Society Meetings.

### NEW YORK ODONTOLOGICAL SOCIETY.

A REGULAR meeting of the New York Odontological Society was held on Tuesday evening, November 15, 1892, at the New York Academy of Medicine, with the President, Dr. Woodward, in the chair.

Dr. Benjamin Lord read a paper entitled "A Brief Review."

(For Dr. Lord's paper, see p. 15.)

### INCIDENTS OF OFFICE PRACTICE.

*Dr. Osborn.*—I hold in my hand two models, the teeth on which are retained in place by means of ordinary copper wire staples. They can be purchased for about twenty cents a pound at any hardware store, and it is almost impossible to break the teeth off, when they are used. They can be passed around for inspection.

Dr. Bödecker then read a paper entitled "The Doctrine of Inflammation."

(For Dr. Bödecker's paper, see page 1.)

### DISCUSSION ON DR. BÖDECKER'S PAPER.

*Dr. Heitzmann.*—I can only be thankful to my friend, Dr. Bödecker, for what he said in his paper about my work. It is certainly remarkable that he scarcely spoke of his own work, and he did a great deal, I am sure, in the study of both the normal and the pathological conditions of the tissues of the teeth. He did not mention that it was himself, in 1878, who first described the reticulum in the dentine and the inflammation of this tissue, which is a rare disease; further, the normal structure of enamel, cementum, the pulp and the pericementum, and the inflammatory changes of all these tissues.

As for myself, I am ignored, forgotten, and abused,—ignored and forgotten in Europe, and abused in this country. The facts represented by Dr. Bödecker are substantiated by means of photography, independent of human eye or human hand. Even the reticulum I had the pleasure of showing to you last season is represented by means of photography. For the past two years a gentleman, Mr. Maximilian Toch, has worked in my laboratory. He is an expert in photography, and succeeds in producing this reticulum and its interconnections in all tissues. My assertion that the animal body is one continuous mass is now proved by him to be true for plants. We know that there are many plants that react upon the least irritation. If the Mimosa pudica is touched by a fly, the whole plant quivers as if convulsed. The doctrine of inflammation I tried to explain twenty years ago, and you have heard what the outcome was. It was ignored. In spite of having a bright and well-known teacher at my side (I refer to Professor S. Stricker), they ignore both of us.

Grawitz greatly surprised me with his assertions at the beginning of this year, where he describes the inflammatory process, and says that the intercellular substance furnishes even more cells than arise from the proliferation of the original or fixed tissue-cells. He bases his assertion upon his own researches as well as those of his pupils, and quite a number of articles have recently been published by him. The strangest thing is the work of Professor Weigert, in Frankfurt. He attacks Grawitz, and calls all his assertions nonsense. Weigert goes so far as to say that the intercellular substance is dead, and could not produce cells. He claims that the cellular pathology is a physical law that cannot be shaken, and this is said by a man who is a thorough adherent of Cohnheim's emigration-theory, who did his best to overthrow the cellular pathology by claiming that there exists no proliferation of the cells, but the image of proliferation is merely due to an emigration of colorless blood-corpuscles, or leucocytes. Is it possible that such things happen in Germany even in our day? I sent my book, with a short letter, to Professor Grawitz, and I had an answer from him in which he admits that he ignored everything I said twenty years ago. My book was published simultaneously in English and German, and the sale of it in Europe was so small that the publishers have reduced the price one-half. No one wants it; it is remarkable, indeed, that the English edition is almost gone, but the German was, financially at least, a failure. Grawitz writes me that in his next publication he will do me full justice.

I said I was abused in this country. I have opponents to my doctrine. This is well enough; every new doctrine has of necessity opponents. We know that an Austrian by the name of Auenbrugger, hundreds of years ago discovered the physical examination of the chest by percussion and auscultation, and said that every physician ought to know the process of making a correct diagnosis of diseases of the cavities of the chest. Woe to the physician to-day who does not know this method of examination. Much the same is my position. A man who discovers novel facts thoroughly contradictory to everything hitherto thought to be true, such as was the cellular pathology, who besides is a little over-enthusiastic, is invariably attacked. If the opponents were honest men and disproved what I said by earnest scientific work, I would not object; but unfortunately it is not so. The opponents use weapons which are not at my disposal, for my conviction is that in matters of this kind only absolute honesty will ever conquer. As long as dishonest means are resorted to for attacking such researches, I am without a weapon; I cannot fight them. I am told that our great bacteriologist, Professor Miller, of Berlin, recently issued a book in which he claims that myself and Dr. Bödecker make assertions regarding the structure of the teeth which no one has ever corroborated. I did not read the sentence, but if Miller said so, he is certainly much mistaken, and I am afraid the source is to be sought in New York. You all know that what I claim, I have demonstrated, and that it was by no means Bödecker alone who publicly announced his concurrence with my own views, but also such men as Frank Abbott, William H. Atkinson, John I. Hart, Roy, Davenport, and a man who never worked in my laboratory, Dr. McCausey, of Janesville, Wisconsin, all of whom have publicly announced that things are as I see them. Still, the assertion is made that it is only myself and Bödecker who make the claim.

But, gentlemen, one salvation exists for me and all my friends, and that is photography. Photo-micrography to-day has made such remarkable progress that we do not need a very expensive apparatus, such as the electric microscope, but an ordinary petroleum lamp is sufficient to produce all the details. Mr. Toch, the gentleman whom I referred to before, is an amateur, and he leaves exposed for hours the plates on which he wants to photograph, to an ordinary photographic light, and the results are marvellous. The details come forth with a distinctness which leaves nothing to be desired. If photographs show all these minutiæ, will there be reason to doubt them ? I hope not, for you know how photographs are produced. The plate is left exposed, and what the plate shows I believe to be true. What is before us? It is a fact that although we have worked hard for the past twenty years, we have received comparatively little acknowledgment for it, and that in Germany they have been just twenty years behind the times. Strange to say that those so-called learned men do not know the history of microscopy and histology, and come forth with discoveries that have been known to us in this country for the past eighteen years. I personally have grown old; my fanaticism is abated. I am still enthusiastic, but not as much so as formerly. I let things come as they will, because I am personally convinced that truth will always conquer, that honesty cannot die. I may die, but the time will come when my views will be victorious. In Germany they will claim that we are built up of individual cells, instead of one mass of living matter. As long as Virchow is at the head of pathological research in Germany, there is no hope for me; and still, if you ask whether I will continue my work, in spite of all these adversities, I say I will, up to the last breath, for I am sure the time will come when this work will be appreciated. It may be late, but the victory is surely ours.

*Dr. Howe.*—I move a vote of thanks to Dr. Bödecker for his able and interesting paper.

Carried.

Adjourned.

John I. Hart, D.D.S.,
*Editor New York Odontological Society.*

